# Establishing the Diagnostic Reference Levels for Common Dubai Health Adult Nuclear Medicine Examinations

**DOI:** 10.3390/life15040649

**Published:** 2025-04-15

**Authors:** Entesar Z. Dalah, Najlaa K. Al Mazrouei, Zahra A. Al Ali

**Affiliations:** 1 Central Diagnostic Imaging Department, Dubai Health, Dubai P.O. Box 2727, United Arab Emirates; 2 College of Medicine, Mohammed Bin Rashid University, Dubai Health, Dubai P.O. Box 2727, United Arab Emirates; 3 Medical Physics Department, Dubai Hospital, Dubai Health, Dubai P.O. Box 2727, United Arab Emirates; 4 Nuclear Medicine Center, Dubai Hospital, Dubai Health, Dubai P.O. Box 2727, United Arab Emirates

**Keywords:** diagnostic reference level, nuclear medicine, computed tomography, radiopharmaceutical, PET/CT, SPECT/CT

## Abstract

Nuclear medicine (NM) procedures are performed using unsealed radioactive sources that are administered to patients, resulting in both internal and external exposure for patients and staff alike. Optimization is mainly concerned with ensuring the use of the lowest sufficient level of radiation to perform a procedure while maintaining adequate image quality. Diagnostic reference levels (DRLs) have been proven effective in aiding optimization in clinical practice. This dose review aims to establish an inclusive DRL system for the common adult NM procedures performed at Dubai Health. Our defined DRLs will focus on both the administered radiopharmaceuticals and the radiation dose metrics derived from hybrid computed tomography (CT). Dose surveys for 1439 adult nuclear medicine procedures performed over twelve months were collected and retrospectively analyzed. DRLs were obtained for a total of eight scintigraphy procedures, four hybrid positron emission tomography procedures with CT (denoted PET/CT), and five target sites for CT hybrid single-photon emission tomography with CT (denoted as SPECT/CT). Our derived DRLs for the scintigraphy, hybrid SPECT/CT and PET/CT procedures are within the reported national DRLs, except for the CT dose of the hybrid SPECT/CT for the neck, abdomen and chest/abdomen sites and the ^18^F PSMA administered activity. A fixed activity dose was administered for a scintigraphy procedure that is weight dependent. This patient dose review serves as a foundational effort aiming to optimize radiation safety and standardize diagnostic practices in NM. Further research is needed to enhance adherence to safety benchmarks.

## 1. Introduction

Nuclear medicine (NM) diagnostic, therapeutic and theragnostic procedures are performed using unsealed radioactive sources that are administered to patients intravenously, via ingestion, or through inhalation, causing internal and external radiation exposure equally to patients and healthcare providers alike. In association with this concern, these practices present the essential need to optimize the radioactivity introduced to a patient [1]. Nuclear medicine physicians, scientists and technologists all share the responsibility and obligation of observing and working to sustain patient radiation safety [2,3,4]. Adopting requirements for patient safety is a consideration recommended by the leading international radiation safety organizations, including, among others, the International Commission on Radiation Protection (ICRP) (Report 135) [5], the International Atomic Energy Agency (IAEA) [6] and the World Health Organization (WHO) [7]. On a national level, the United Arab Emirates (UAE) Federal Authority for Nuclear Regulation (FANR) enforces and regularly amends patient radiation safety guidelines [8,9].

Fundamentally, dose optimization can be achieved using the conceptual guidelines of the diagnostic reference levels (DRLs), which are derived based on patient radiation exposure in clinical examinations [5]. DRLs enable comparisons of the doses selected for a given procedure alongside existing reported DRLs, and the identification of procedures or healthcare centers in need of dose reduction action.

Estimating a guideline radiation dose level, i.e., DRL, received by a population is vital, not only in providing enough knowledge to outweigh the radiation-induced risks, but also to ensure that the procedure undertaken is justified. For specific NM clinical exams, the ideal DRL dose quantity is the level of radioactivity administered as a function of body weight, as recommended by ICRP Report 135 [5]. Radioactivity administered as a function of weight, measured in Bq/kg, is a determination accepted for use in children, adolescents and non-obese adults [5]. For a very obese patient, a fixed radioactivity dose measured in Bq is acceptable, per ICRP Report 135 [5]. For other NM exams in which the injected radioactivity is dominantly trapped in a fixed organ such as the thyroid, a fixed radioactivity (weight independent) determination is also acceptable [5]. For NM procedures conducted using hybrid scan modalities such as hybrid single-photon emission computed tomography (SPECT) with computed tomography (CT) (denoted as SPECT/CT) and hybrid positron emission tomography (PET) with CT (denoted as PET/CT), DRLs should be set for each modality independently, i.e., separate DRLs for the injection activity relating to SPECT or PET, and DRLs for the CT. The dose quantities recommended for the establishment of DRLs for CT are the volume CT dose index (CTDI_vol_), measured in mGy, and the dose length product (DLP), measured in mGy.cm.

This health sector patient dose report is the first review focusing on our governmental Nuclear Medicine Center in the Emirate of Dubai, which operates under the umbrella of Dubai Health. An initial slate of national UAE NM DRLs (iNDRLs) was proposed in 2015 and published in 2018 [10]. The iNDRLs were limited to reporting the radioactivity associated with the common adult general NM procedures, i.e., procedures using gamma cameras only. The present patient dose review aims to establish an inclusive guideline DRL system for the most common scintigraphy NM procedures (those using PLANAR and SPECT techniques) and hybrid NM (using SPECT/CT and PET/CT) procedures performed for adult patients. For quality assurance and internal auditing, the guideline DRLs proposed will use an adult of average size as a benchmark.

The organization of this paper places the literature review in Section 1 (Introduction), while the methodology is covered in Section 2 (Materials and Methods), the study observation and findings are presented in Section 3 (Results), a discussion of the findings and limitations encountered is presented in Section 4 (Discussion), and, finally, we state the study’s conclusions in Section 5.

## 2. Materials and Methods

Patient radioactivity and CT dose data used in this healthcare sector report were approved by the Dubai Scientific Research Ethics Committee (DSREC) at Dubai Health (14 August 2024). Patients’ radioactivity and CT dose survey data were collected from 1 January to 31 December 2024. The radioactivity and CT dose datasets were arbitrarily retrieved for a retrospective analysis to determine the common scintigraphy and hybrid NM procedures performed for adults at our dedicated nuclear medicine center. The electronic patient radiation dose monitoring and tracking system of DOSE TQM (Qaelum NV) [11] was used to collect the relevant patient radioactivity and CT dose survey data. The facility is equipped with two gamma cameras, specifically the GE Discovery 630 NM (GE Healthcare, Waukesha, WI, USA), a SPECT/CT scanner (GE Discovery 670, 16 slices (GE Healthcare, Waukesha, WI, USA)), and a PET/CT scanner (GE Discovery MI DR, 64 slices (GE Healthcare, Waukesha, WI, USA)). Patient demographic and procedure information including age, weight, study description, body part examined, radiopharmaceuticals, NM modality and CT scan acquisition parameters were also collected using DOSE TQM. Here, patients aged 15 years and older were considered adults.

For the gamma cameras and SPECT/CT system, Technetium (Tc-99m/^99m^Tc) is the predominant isotope, which is combined with various pharmaceuticals to produce specific radiopharmaceuticals. These include the following: ^99m^Tc-hydroxy diphosphonate (HDP) for bone scans; ^99m^Tc-methoxyisobutylisonitrile (MIBI) for myocardial imaging (including rest or stress) and parathyroid assessments; ^99m^Tc-Pertechnetate for thyroid imaging; ^99m^Tc-dimercaptosuccinic acid (DMSA) for static renal scans; ^99m^Tc-mercaptoacetyltriglycine (MAG3) for dynamic renal evaluations (renograms), ^99m^Tc-colloid for gastrointestinal studies; and ^99m^Tc-pyrophosphate (PYP) for cardiac assessments. Scintigraphy images are based on collecting counts that have been absorbed in an organ. Scintigraphy images can be acquired as PLANAR or SPECT, using a gamma camera (Discovery NM 630, GE Healthcare, Waukesha, WI, USA). The difference lies in the data acquisition technique. PLANAR is when a gamma camera is used to acquire a 2D image based on collecting counts acquired from a single 2D image or from a combined opposite acquired pair of 2D images. In contrast, SPECT images are also acquired using a gamma camera, but this time the collected counts are acquired from multiple 2D images using multiple angles. For detailed information on the difference between PLANAR vs. SPECT images, the reader is referred to [12]. In contrast, the primary isotope utilized in PET/CT is ^18^F-fluorodeoxyglucose (FDG), specifically for comprehensive oncology evaluations that encompass the whole body (WB) or extend from the skull to mid-thigh (SMT). Additional isotopes used in PET/CT include Gallium-68 DOTATOC (^68^Ga-DOTATOC) for neuroendocrine tumor diagnosis, ^18^F-fluorocholine for parathyroid imaging, and ^18^F-prostate-specific membrane antigen (PSMA) for prostate cancer imaging.

The DRL value, for a specific NM procedure, which represents our sector will be set at the median distribution of the scintigraphy and hybrid common NM procedures. In addition, patient dose indicators will be presented in the 75th percentile for all CT dose indices and NM radioactivity doses [5,13,14]. For quality assurance, optimization and internal auditing purposes, we considered a standardized body size (60–80 kg) as per [5].

### Statistical Analysis

Statistical analysis was conducted using GraphPad Prism 8, V8.03, GraphPad Software, San Diego, CA, USA. Quantitative variables are expressed as the median and 75th percentiles. The Pearson coefficient test was used for correlation analysis. *p* = 0.05 was used for significance.

## 3. Results

### 3.1. Survey Sample

Dose surveys for a total of 1439 (495 scintigraphy including the 2 gammas and SPECT/CT and 944 PET/CT) adult nuclear medicine procedures performed over a 12-month period were collected and retrospectively analyzed. The most common scintigraphy NM procedure registered for adults over the stated period was thyroid imaging using ^99m^Tc-pertechnetate (sample size,128) followed by myocardial (rest or stress) using ^99m^Tc MIBI (sample size, 121), renogram scan using ^99m^Tc MAG3 (sample size, 84), WB bone scan using ^99m^Tc-HDP (sample size, 60), parathyroid using ^99m^Tc MIBI (sample size, 34), gastro emptying using ^99m^Tc colloid (sample size, 26) and cardiac using ^99m^Tc PYP (sample size, 15). The most common PET/CT procedure performed was WB/SMT using ^18^F-FDG (sample size, 662) followed by ^18^F PSMA (sample size, 131), ^68^Ga DOTATOC (sample size, 54) and ^18^F CHOLINE parathyroid (sample size, 18).

### 3.2. CT Acquisition Parameters

For the CT of the hybrid SPECT/CT and PET/CT, the machine default acquisition parameters for the different regions are demonstrated in Table 1 and Table 2, respectively.

Limited studies disclose the CT scan acquisition parameters used for the hybrid scans whether SPECT/CT or PET/CT. Allkhybari and colleagues [15] shared the CT scan acquisition parameters associated with the different CT studies of the hybrid PET/CT. An example is the WB CT scan; our scan acquisition parameters for the WB CT of the hybrid PET/CT are in line with the CT scan acquisition parameters reported in [15]. A slight difference was noticed in the maximum tube current (mA) and the pitch reported in [15] compared to our default setup. Our maximum end of mA is higher (300 vs. 244) and our pitch is lower (1.375 vs. 1.675). The higher the mA, the higher the exposure, and the lower the pitch, the higher the dose overlap [16]. However, since smart mA is enabled, in Table 1 and Table 2, mA has been adjusted whenever there is a need to compensate for high attenuation offering an optimized mA. For CT acquisition parameters used to perform diagnostic CT exams, the readers are referred to [17].

### 3.3. Weight Impact

#### 3.3.1. Scintigraphy Procedures

**Figure 1:**
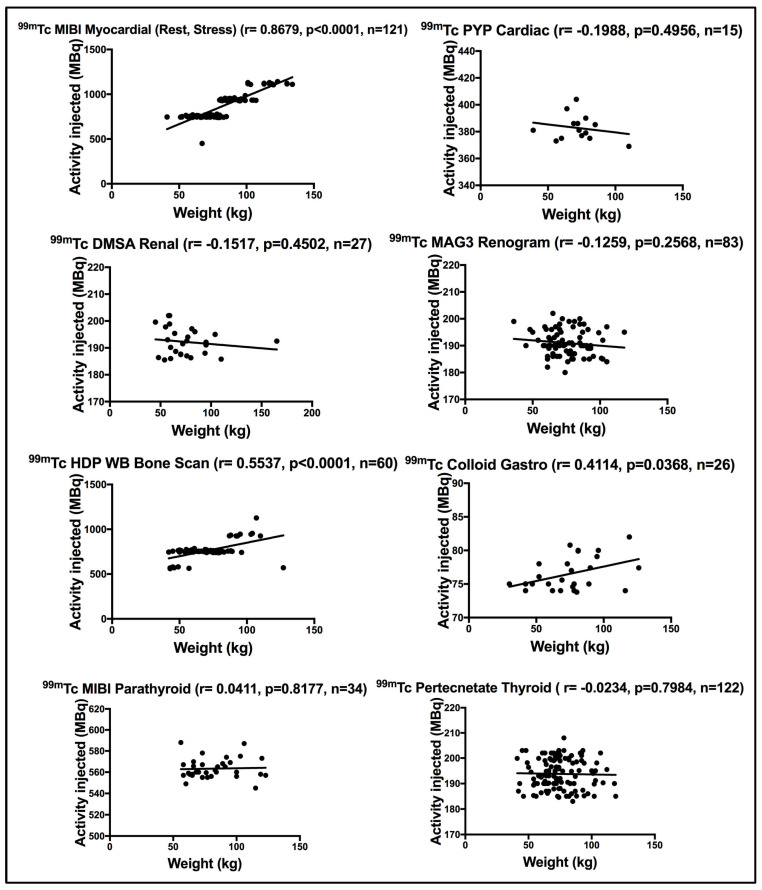
Correlation between weight and the administered activity for patients subjected to scintigraphy nuclear medicine procedures. r denotes the Pearson correlation coefficient, *p* < 0.05 denotes significance, and n denotes the sample size.

Using the Pearson correlation coefficient test, a positive strong significant correlation was seen between patient weight and the administered activity for patients subjected to myocardial (rest or stress) using ^99m^Tc MIBI myocardial (r = 0.8679, *p* < 0.0001). ^99m^Tc HDP WB bone scan showed a moderately significant correlation (r = 0.5537, *p* < 0.0001). The moderate correlation seen implies that the activity administered was not weight specific. Figure 1 demonstrates a fixed ^99m^Tc HDP administered activity for most of the patients between 50 and 100 kg enrolled for WB bone scan. No correlation was seen between patient weight and the administered activity for patients who underwent ^99m^Tc MIBI parathyroid (r = 0.0411, *p* = 0.8177) and ^99m^Tc Pertechnetate thyroid image (r = −0.0234, *p* = 0.7984). This is expected as the administered activity is trapped in a single organ, i.e., the thyroid. A poor correlation was seen for patients subjected to renal static scan using ^99m^Tc DMSA (r = −0.1517, *p* = 0.4502), renogram scan using ^99m^Tc MAG3 (r = −0.1259, *p* = 0.2568) and cardiac scan using ^99m^Tc PYP (r = −0.1988, *p* = 0.4956). ^99m^Tc colloid gastro scan disclosed a moderately significant correlation with patient weight (r = 0.4114, *p* = 0.0368) using the Pearson correlation coefficient test. Figure 1 demonstrates the correlation between weight and the administered activity for patients subjected to scintigraphy (both PLANAR and SPECT) procedures. Table 3 defines the scan technique (PLANAR, SPECT or both) used to perform each scintigraphy scan.

#### 3.3.2. PET Procedures

**Figure 2:**
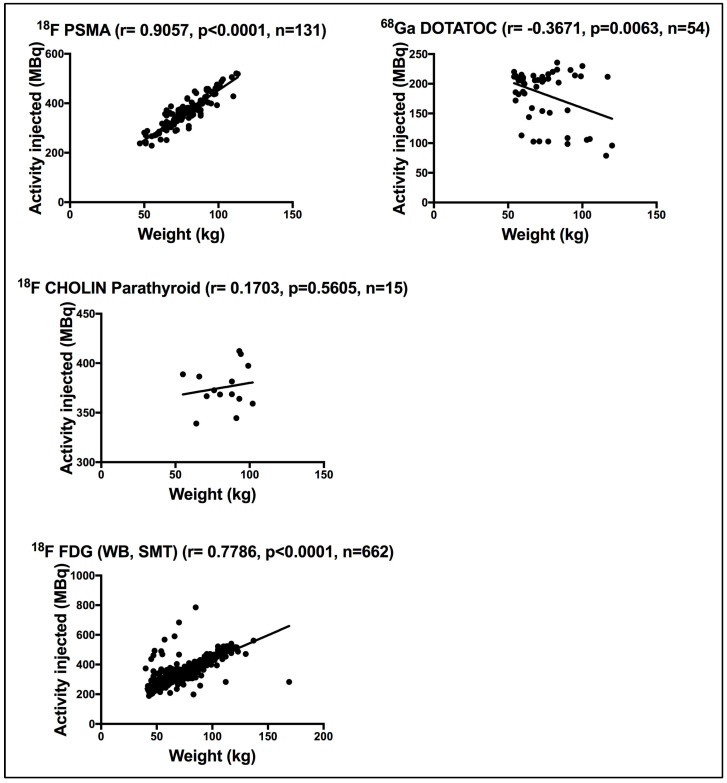
Correlation between weight and the administered activity for patients subjected to PET/CT nuclear medicine procedures. r denotes the Pearson correlation coefficient, *p* < 0.05 denotes significance, and n denotes the sample size.

Using the Pearson correlation coefficient test, a strong significant correlation was seen between patient weight and the administered activity for patients subjected to ^18^F PSMA (r = 0.9057, *p* < 0.0001) and ^18^F-FDG WB/SMT (r = 0.7786, *p* < 0.0001). The ^68^Ga DOTATOC procedure disclosed a moderate significant correlation (r = −0.3671, *p* = 0.0063). Finally, the ^18^F CHOLIN parathyroid scan showed a poor insignificant correlation (r = 0.1703, *p* = 0.5605) between weight and the administered activity. Figure 2 demonstrates the correlation between weight and the administered activity for patients subjected to PET procedures.

### 3.4. CTDI_vol_ of the Hybrid CTs

**Figure 3:**
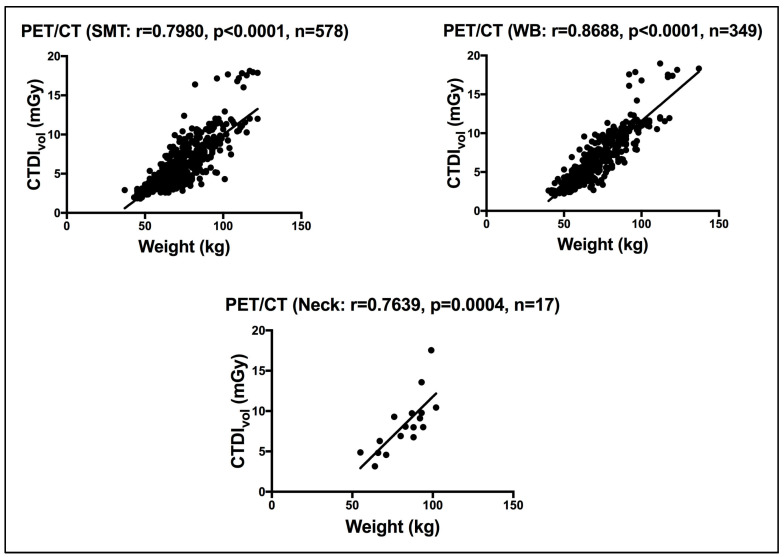
Correlation between weight and the CTDI_vol_ for patients scanned on the CT of the hybrid PET/CT. r denotes the Pearson correlation coefficient, *p* < 0.05 denotes significance, and n denotes the sample size.

For the hybrid CT of the PET/CT, a strong significant correlation was seen between weight and the CTDI_vol_, for patients subjected to WB, SMT and neck CT scans, using a Pearson correlation coefficient test, as shown in Figure 3. WB and SMT CT scans include the torso all the way to the pelvis region. The CTDI_vol_ in this site of the body is known to be affected by weight, unlike the neck which is known to be more age dependent [5]. Using linear regression analysis, Al Shurbaji et al. [24] studied the impact of CT scan acquisition and patient-related factors on CT dose, reporting a significant correlation between patient weight and CTDI_vol_ across their investigated CT exams (chest, cardiac, abdomen and pelvis).

**Figure 4:**
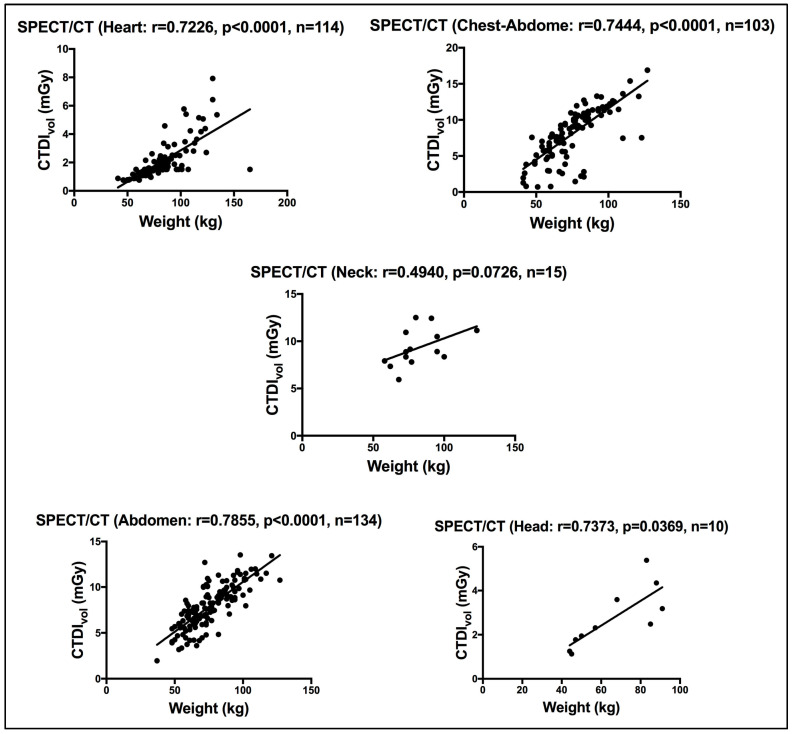
Correlation between weight and the CTDI_vol_ for patients scanned on the CT of the hybrid SPECT/CT. r denotes the Pearson correlation coefficient, *p* < 0.05 denotes significance, and n denotes the sample size.

For the hybrid CT of the SPECT/CT procedures, the CT is performed for targeted body sites. Here, we report the CT doses for five target body sites (head, neck, heart, abdomen and chest/abdomen). While a strong significant correlation was seen between patient weight and the CTDI_vol_ for the abdomen, chest/abdomen and heart sites, a moderate insignificant correlation was seen for the neck site using the Pearson correlation coefficient test. Figure 4 shows the correlation between weight and the CTDI_vol_ for all five target body sites performed with hybrid SPECT/CT. Like [25], CT in our practice is acquired mainly for localization (L) and attenuation correction (AC), unlike [14], where CT was also used for diagnosis purposes.

### 3.5. Diagnostic Reference Levels, DRLs

#### 3.5.1. DRLs for the Administered Activity for Scintigraphy and PET Procedures

Median values were taken as representatives of the DRLs for the common nuclear medicine procedures at our nuclear medicine facility as recommended by Vañó et al. [5]. The health sector patient activity survey results (50th and 75th percentiles) were determined for each radiopharmaceutical used for scintigraphy procedures, as shown in Table 3. For scintigraphy procedures where the administered activity strongly correlates with patient weight, the proposed DRL is presented for the average-sized adult (60–80 kg) and for the whole cohort enrolled.

Table 4 demonstrates the radiopharmaceuticals used for PET/CT procedures. Like scintigraphy, the activity survey results (50th and 75th percentiles) were determined for each radiopharmaceutical used. For PET radiopharmaceuticals where the administered activity strongly correlates with patient weight, the proposed DRL is presented for the average-sized adult (60–80 kg) and for the whole cohort enrolled.

#### 3.5.2. DRLs for CTDI_vol_ and DLP in the Hybrid CT Scans

The health sector patient CT dose survey results (50th and 75th percentiles) were determined for each target site, as shown in Table 5 for hybrid SPECT/CT and Table 6 for hybrid PET/CT.

## 4. Discussion

To ensure the highest standard of care in radiology and nuclear medicine, we must adhere to the three pillars of radiation protection, namely justification, optimization and limitation [28,29]. Optimization is particularly concerned with ensuring the use of the lowest radiation to perform a procedure while maintaining a diagnosable image. DRLs have been proven effective in optimizing radiation exposure in clinical setups despite the radiological modality [30,31,32,33]. It is important to clarify that DRLs are not dose limits nor a constraint, yet observing DRLs results in superior quality of healthcare service and good practice [5]. DRLs aim to identify and address areas of deficiency within the practice, including department protocols, procedures and equipment configuration and performance [27]. It can also help detect areas with knowledge gaps such as patient positioning and open scout, especially in CT given its vital impact on the resulting CT dose [34,35,36].

Herein, we report the first comprehensive dose guide (DRLs) for adults subjected to scintigraphy (both PLANAR and SPECT), hybrid PET/CT and the CT of the hybrid SPECT/CT procedures within the Dubai Health sector. DRLs were established for a total of eight scintigraphy procedures (PLANAR and SPECT), four hybrid PET/CT procedures and five target sites for the CT of the hybrid SPECT/CT. DRLs were established for both the administered radioactivity doses as well as for both CT dose indices (CTDI_vol_ and DLP). The present work reports the DRLs based on the entire sample size enrolled in each NM procedure as well as for a standard-size adult (60–80 kg), allowing better dose optimization [5]. Such a dose guide will assist in delivering a safe healthcare service by permitting optimization and internal auditing.

In nuclear medicine, CT dose exposure differs and varies according to the type of examination, scan region, and scan range. In this survey, the CT-established DRLs for the hybrid PET/CT were based on the clinical procedure; this is in line with the UK and Australian national reported DRLs [20,25], respectively. However, for the hybrid SPECT/CT, the CT DRLs were reported based on the target site such as the head, neck, chest, abdomen, etc., which is in line with the Kuwait and Japan national reported DRLs [14,27].

Figure 1 demonstrates the wide variation in the administered activities registered for ^99m^Tc HDP WB bone, with a clear dispersion of activity for the same weight being evident. Clearly, a fixed activity was administered for ^99m^Tc HDP WB bone for patients’ weight ranging from 50 to 100 kg. It is also evident in Figure 1 that different ^99m^Tc MIBI myocardial activities are administered to patients of the same weight. Ideally, the administered activity should be defined based on MBq/kg to meet detector sensitivity, particularly for non-thyroid organ procedures. Figure 2 illustrates the strong correlation between ^18^F FDG administered activity and weight for patients subjected to WB/SMT, implying that the administered activity is weight dependent. Nonetheless, cases of twice the administered activity for the same weight were evident.

Radiation doses should be minimized while ensuring enough diagnostic information is obtained. It is crucial to maintain clinically acceptable limits, avoiding both too-high and too-low doses. While there is a minimum dose required for effective diagnostics, increasing radiation levels can enhance quality only to a certain point, after which further increases result in a degraded image quality [37,38].

The present work encountered some limitations. Primarily, image quality evaluations were not part of the present dose review report. Not all scintigraphy (PLANAR and SPECT), hybrid SPECT/CT and hybrid PET/CT procedures performed in our dedicated nuclear medicine facility were included in the present report due to the small survey samples. Theragnostic procedures were not included in the present work, in part due to the insufficient sample size and the need for better labeling and structuring for such procedures. Another fact that is associated with theragnostic procedures is the fact that the injected activity depends primarily on patient’s disease history and stage. Hence, establishing a DRL based on the theragnostic procedure name is not appropriate nor accurate. Aligning our derived DRLs for the CT of the hybrid PET/CT with the nationally reported DRLs remains a challenge because of the inconsistent style in reporting the CT DRLs. Where some report DRLs based on the procedure [20,25,26], others report based on the target site [14,27,39].

A particular pressing challenge is the substantial wide observed variation across national and existing DRL records for a specific hybrid CT and radiopharmaceutical examination, for example, the CTDI_vol_ of the hybrid SPECT/CT for the neck site, with CTDI_vol_ reporting DRLs ranging from as low as 4.50 mGy up to 7.20 in Kuwait and Australia [20,27], respectively. On a similar note is the DLP of the hybrid SPECT/CT for the neck site, with DLP-reported DRLs ranging from 199 up to 240 mGy.cm in [20,26]. The ^18^F FDG WB/SMT reported DRLs range from as low as 258 up to 370 MBq in Qatar and Korea [18,22], respectively. Similarly, in the ^99m^Tc MIBI parathyroid administered activity, the DRLs reported ranged from 384 up to 800 MBq in Qatar and Australia/Japan [15,18,20]. Further, given that the data obtained was restricted to the single governmental center of nuclear medicine in the Emirate of Dubai, different DRL baselines are expected to vary across the private institutes, leading to different national DRLs. Still, our findings highlight the need for continued effort in promoting protocol optimization and standardization to allow for improvements. Work is needed to reduce the CT dose (both CTDI_vol_ and DLP) for the neck, abdomen and chest/abdomen site for the hybrid SPECT/CT. Our derived DRLs for ^18^F PSMA administered activity are almost twice the DRL administered activity reported in Saudi Arabia [15] for the average-sized adult (341 vs. 184 MBq, respectively). Future work will focus on reviewing the common nuclear medicine procedures for pediatric in Dubai Health and setting the DRLs based on age and weight bands following [5,40].

## 5. Conclusions

Our derived DRLs, whether for the scintigraphy (PLANAR and SPECT), hybrid SPECT/CT or PET/CT procedures, are within the national reported DRLs, except for the CT dose of the hybrid SPECT/CT for the neck, abdomen and chest/abdomen sites and the ^18^F PSMA administered activity. This patient dose review serves as a foundational effort to optimize radiation safety and standardize diagnostic practices in NM. Further research is needed to enhance adherence to safety benchmarks.

## Figures and Tables

**Table 1:** CT acquisition parameters for the CT of the hybrid SPECT/CT.

Study	kV	Smart mA	Rotation Time (s)	Pitch	Slice Thickness (mm)	Speed (mm/Rotation)	Total Exposure Time (s)	Detector Rows
Neck	120	20–440	0.5	1.375	5.0	13.75	15.34	16
Head	120	20–440	0.5	1.350	5.0	13.50	15.63	8
Heart	120	10–440	0.8	0.938	5.0	18.75	18.47	16
Abdomen/Chest-Abdomen	120	20–440	0.5	1.375	5.0	27.50	7.91	16

Tube potential kilovoltage (kV); tube current (mA); seconds (s).

**Table 2:** CT acquisition parameters for the CT of the hybrid PET/CT.

Study	kV	Smart mA	Rotation Time (s)	Pitch	Slice Thickness (mm)	Speed (mm/Rotation)	Total Exposure Time (s)	Detector Rows
SMT	120–140	30–300	0.8	1.375	5.0	55.0	15.11	40
WB	120–140	30–300	0.8	1.375	5.0	55.0	27.10	40
Head and Neck	120	30–300	0.8	0.984	3.75	39.37	-	40

Skull to mid-thigh (SMT); whole body (WB); tube potential kilovoltage (kV); tube current (mA); seconds (s).

**Table 3:** Radiopharmaceutical activity review and DRLs for scintigraphy procedures.

Procedure and Radiopharmaceutical	Scan Acquisition	Age, Year (Minimum–Maximum)	Weight, kg	Sample Size	Activity Injected, MBq		International References, MBq
					Median (DRL)	75TH PER	
Myocardial (Rest or Stress) and ^99m^Tc MIBI	SPECT	28–90	41–134 ^+^	121	767	940	926 Qatar [18], 650 France [19], 600 Australia [20], 750: rest, 550: stress Lithuania [21]
			60–80	56	752	761	880 Japan [14],
Cardiac and ^99m^Tc PYP	PLANAR+SPECT	27–87	39–110 ^-^	15	381	388	800 Japan [14],
Bone scan and ^99m^Tc HDP	PLANAR+SPECT ^++^	15–77	42–127 *	60	755	767	740 Qatar [18], 925 Korea [22], 670 France [19], 900 Australia [20], 950 Japan [14], 658 Irish [23],
Gastro Emptying and ^99m^Tc Colloid	PLANAR	24–86	30–126 *	26	75	78	36 Qatar [18], 111 Korea [22], 40 Australia [20],
Renal (static scan) and ^99m^Tc DMSA	PLANAR ***	16–87	45–165 ^-^	27	192	196	101 Qatar [19], 185 Korea [22], 210 Japan [14], 84 [Irish [23],
Renogram (dynamic scan) and ^99m^Tc MAG3	PLANAR	16–82	36–118 ^-^	84	190	195	189 Qatar [18], 500 Korea [22], 180 France [19], 300 Australia [20], 380 Japan [14], 105 Irish [23], 100 Lithuania [21]
Thyroid Imaging and ^99m^Tc Pertechnetate	PLANAR	15–78	41–119 **	128	193	200	195 Qatar [18], 217 Korea [22], 110 France [19], 200 Australia [20], 300 Japan [14], 100 Irish [22],
Parathyroid and ^99m^Tc MIBI	PLANAR+SPECT ^++^	31–87	56–123 **	34	560	568	384 Qatar [18], 740 Korea [22], 800 Australia [20], 800 Japan [14], 740 Irish [23], 740 Lithuania [21]

^+^ Strong correlation between activity injected and weight using Pearson coefficient test; * moderate correlation between activity injected and weight using Pearson coefficient test; ^-^ poor correlation between activity injected and weight using Pearson coefficient test; ** no correlation between activity injected and weight using Pearson coefficient test; ^++^ sometimes only PLANAR; *** SPECT if needed, but it is very rare.

**Table 4:** Radiopharmaceutical activity review and DRLs for PET procedures.

Procedure and Radiopharmaceutical	Age, Year (Minimum–Maximum)	Weight, kg	Sample Size	Activity Injected, MBq		International References, MBq
				Median (DRL)	75TH PER	
Oncology (whole body, WB/skull- to mid-thigh, SMT) and ^18^F FDG	16–92	40–169 ^+^	662	343	381	258 Qatar [18], 370 Korea [22], 260 France [19], 270 Australia [20], 334 Australia [22], 500 Lithuania [21]
		60–80	339	334	358	368 Irish [23], 307 Saudi Aribia [15]
Oncology (Prostate) and ^18^F PSMA	44–99	47–113 ^+^	131	363	499	-
		60–80	65	341	362	184 Saudi Aribia [15]
Endocrine and ^68^Ga DOTATOC	15–74	54–120 ^-^	54	206	212	200 Australia [20], 172 Saudi Aribia [15]
Parathyroid and ^18^F CHOLIN	27–95	55–102 ^-^	18	369	391	-

^+^ Strong correlation between activity injected and weight using Pearson coefficient test; ^-^ poor correlation between activity injected and weight using Pearson coefficient test.

**Table 5:** CT dose review and DRLs for hybrid CT of SPECT/CT.

Target Body Part/Purpose of Use	Weight, kg	Sample Size	CTDI_vol_, mGy		DLP, mGy.cm		International References	
			Median (DRL)	75TH PER	Median (DRL)	75TH PER	CTDI_vol_, mGy	DLP, mGy.cm
Neck/AC and L	58–123 **	14	8.90	11.00	380	451	5.80 ^-^ Japan [14], 7.2 Australia [20], 5.90 UK [24], 5.46 Irish [22], 5.9 [26], 4.5 ^-^ Kuwait [27]	210 ^-^ Japan [14], 240 Australia [20], 210 UK [24], 124 Irish [22], 199 [26], 181 ^-^ Kuwait [27]
Head/AC and L	44–91 **	10	2.40	3.78	96	158	-	-
Heart/AC and L	41–165	114	1.57	2.36	42	60	-	-
	60–80	49	1.47	1.60	35	47	4.50 Japan [14], 2.2 [26], 2.1 UK [24], 4.26 Qatar [18]	180 Japan [14], 53 [26], 36 UK [24], 104 Qatar [18]
Abdomen/AC and L	37–127	134	7.82	9.71	500	716	5.00 Japan [14]	210 Japan [14]
	60–80	61	7.20	8.11	438	600	-	-
Chest-Abdomen/AC and L	41–127	103	8.77	10.84	641	860	4.10 * Japan [14]	170 * Japan [14]
	60–80	44	8.10	9.51	631	775	4.86 Qatar [18]	211 Qatar [18]

Attenuation correction (AC); localization (L); ** weight independent; * chest only; ^-^ head and neck.

**Table 6:** CT dose review and DRLs for hybrid CT of PET/CT.

Target Body Part/Purpose of Use	Weight, kg	Sample Size	CTDI_vol_, mGy		DLP, mGy.cm		International References	
			Median (DRL)	75TH PER	Median (DRL)	75TH PER	CTDI_vol_, mGy	DLP, mGy.cm
Neck, AC and L	55–102 **	17	8.00	9.75	676	805	-	-
Whole body, WB/AC and L	40–137	349	6.35	9.26	748	1070	-	-
	60–80	171	6.11	7.36	700	892	6.1 Japan [14], 5.7 Irish [23], 4.1 Kuwait [13], 2.9 [26]	600 Japan [14], 665 Irish [23], 684 Kuwait [13], 310 [26]
Skull to mid-thigh, SMT/AC and L	37–122	578	5.73	8.0	660	948	-	-
	60–80	298	5.11	6.40	561	766	4.20 and 5.3 Australia [20], 5.30 Qatar [18]	430 and 555 Australia [20], 548 Qatar [18]

Attenuation correction (AC); localization (L); ** weight independent.

## Data Availability

Data are contained within the article.

## Abbreviations

NM	(nuclear medicine)
DRL	(diagnostic reference level)
CT	(computed tomography)
PET	(positron emission tomography)
SPECT	(single-photon emission tomography)
^18^F	(Fluorine-18)
^18^F PSMA	(^18^F-prostate-specific membrane antigen)
ICRP	(International Commission on Radiological Protection)
IAEA	(International Atomic Energy Agency)
WHO	(World Health Organization)
UAE	(United Arab Emirates)
FANR	(Federal Authority for Nuclear Regulation)
CTDI_vol_	(volume Computed Tomography Dose Index)
DLP	(Dose Length Product)
iNDRLs	(initial National DRLs)
DSREC	(Dubai Scientific Research Ethics Committee)
DOSE TQM	(Electronic patient radiation dose monitoring and tracking system)
GE	(General Electric)
DICOM	(Digital imaging and communication in medicine)
Tc-99m	(Technetium- 99m)
HDP	(Hydroxydiphosphonate)
MIBI	(Methoxyisobutylisonitrile)
DMSA	(Dimercaptosuccinic Acid)
MAG3	(Mercaptoacetyltriglycine)
PYP	(Pyrophosphate)
FDG	(fluorodeoxyglucose)
WB	(whole body)
SMT	(skull to mid-thigh)
^68^Ga	(Gallium-68)
DOTATOC	(Edotreotide (USAN, also known as (DOTA^0^-Phe^1^-Tyr^3^) octreotide, a somatostatin receptor targeted ligand)
kVp	(Peak kilovoltage)
mAs	(tube current)
AC	(attenuation correction)
L	(localization)
Bq	(Becquerel)
MBq	(MegaBecquerel)
mGy	(Milligray)
